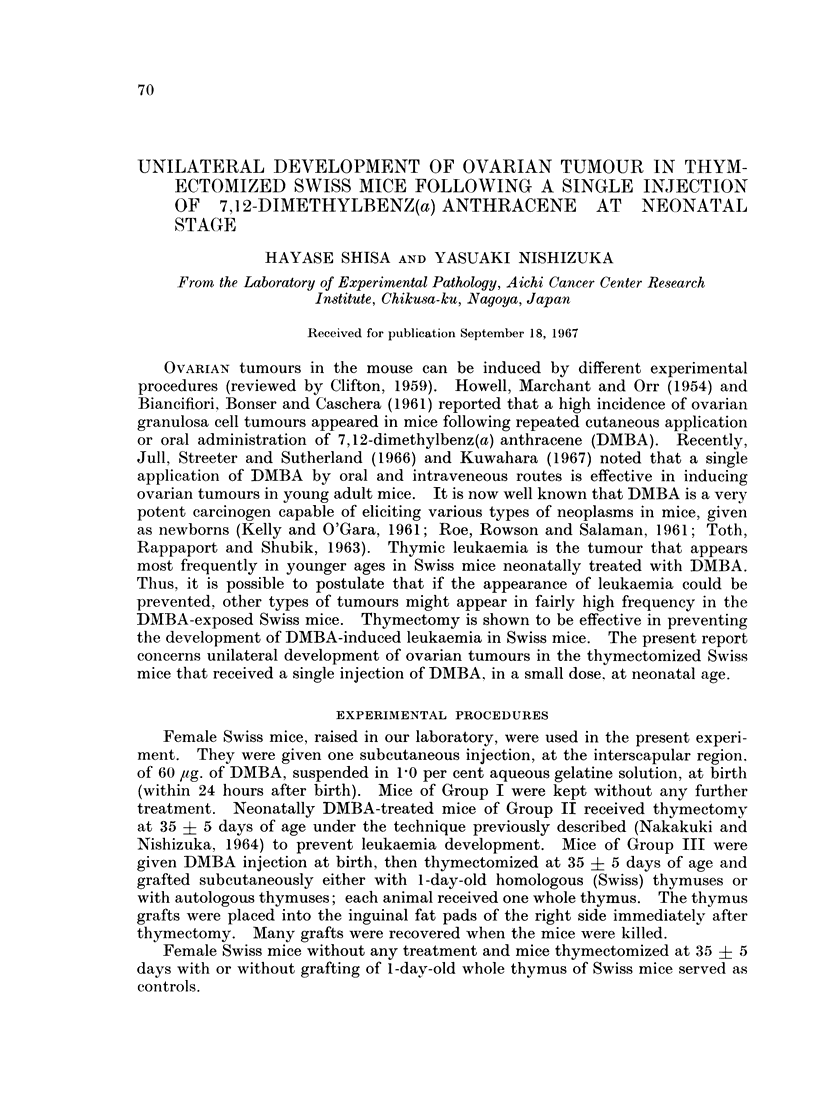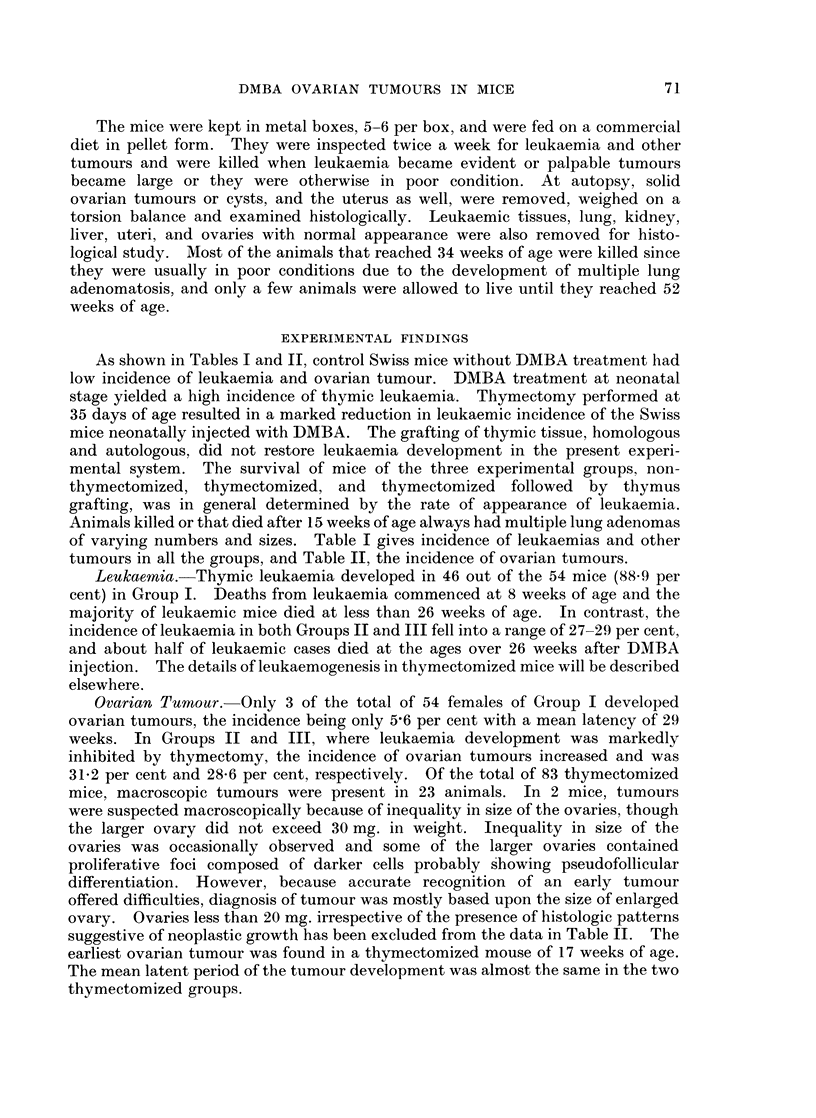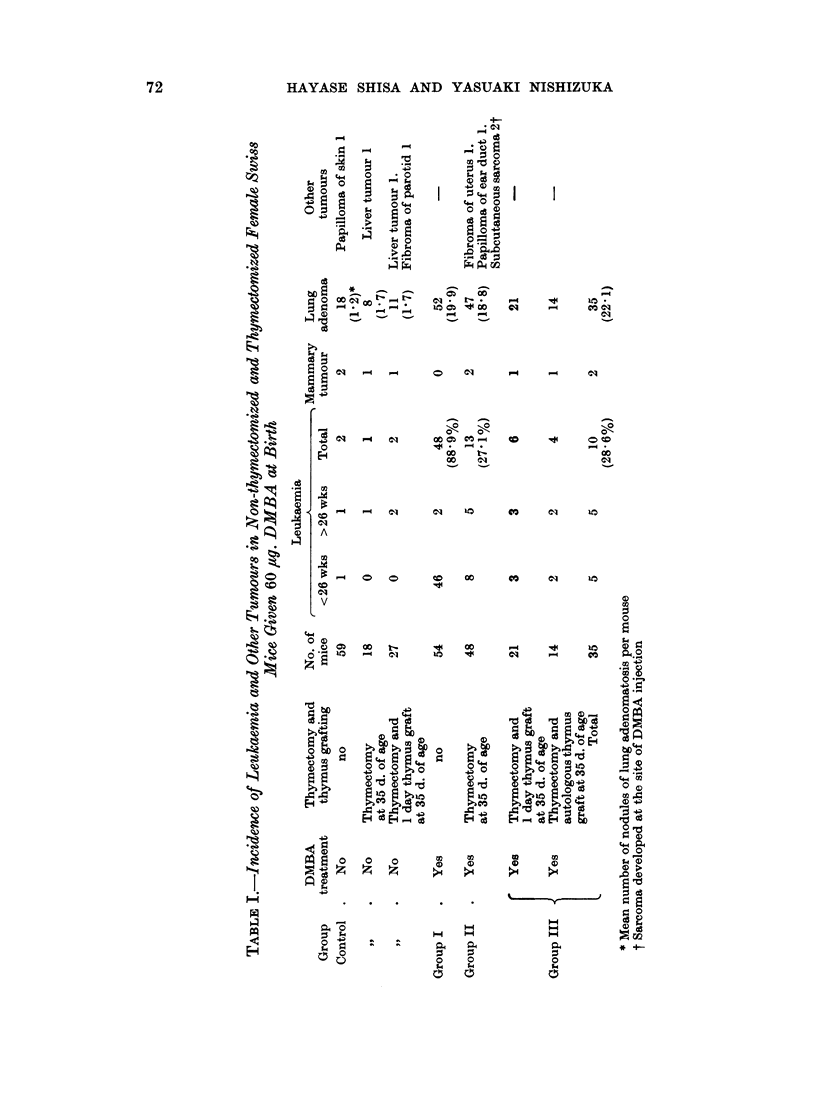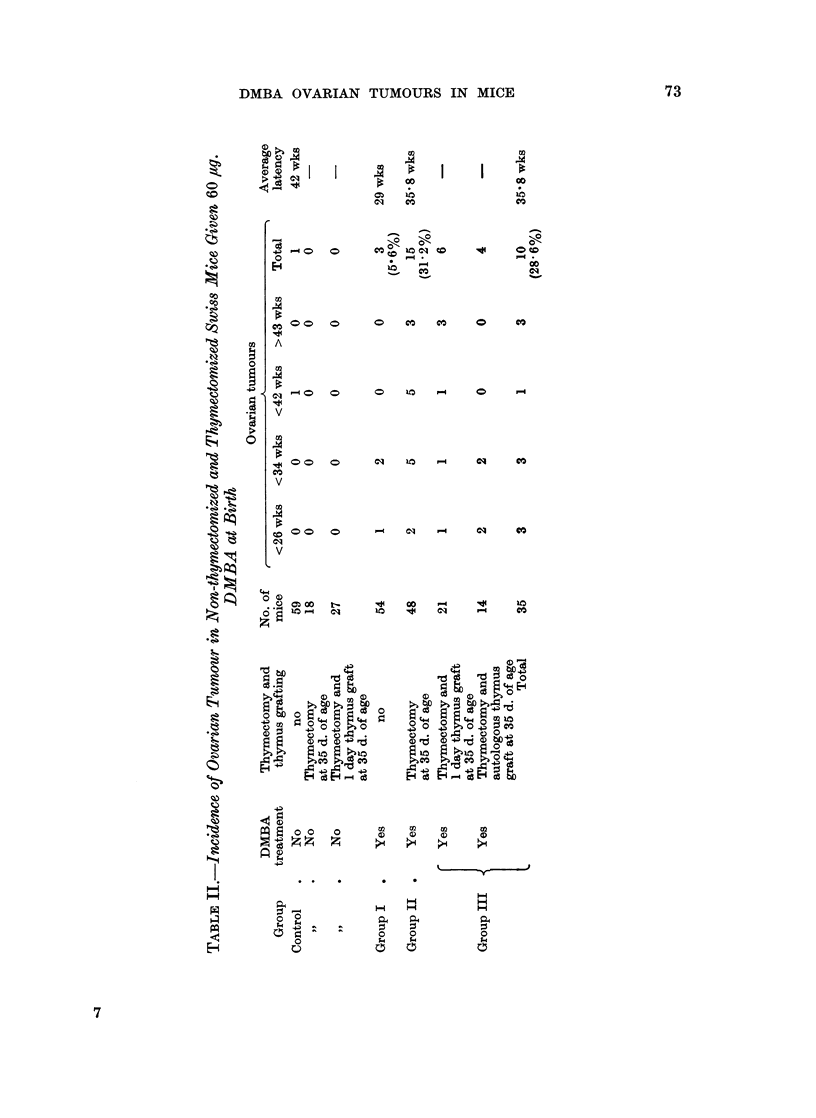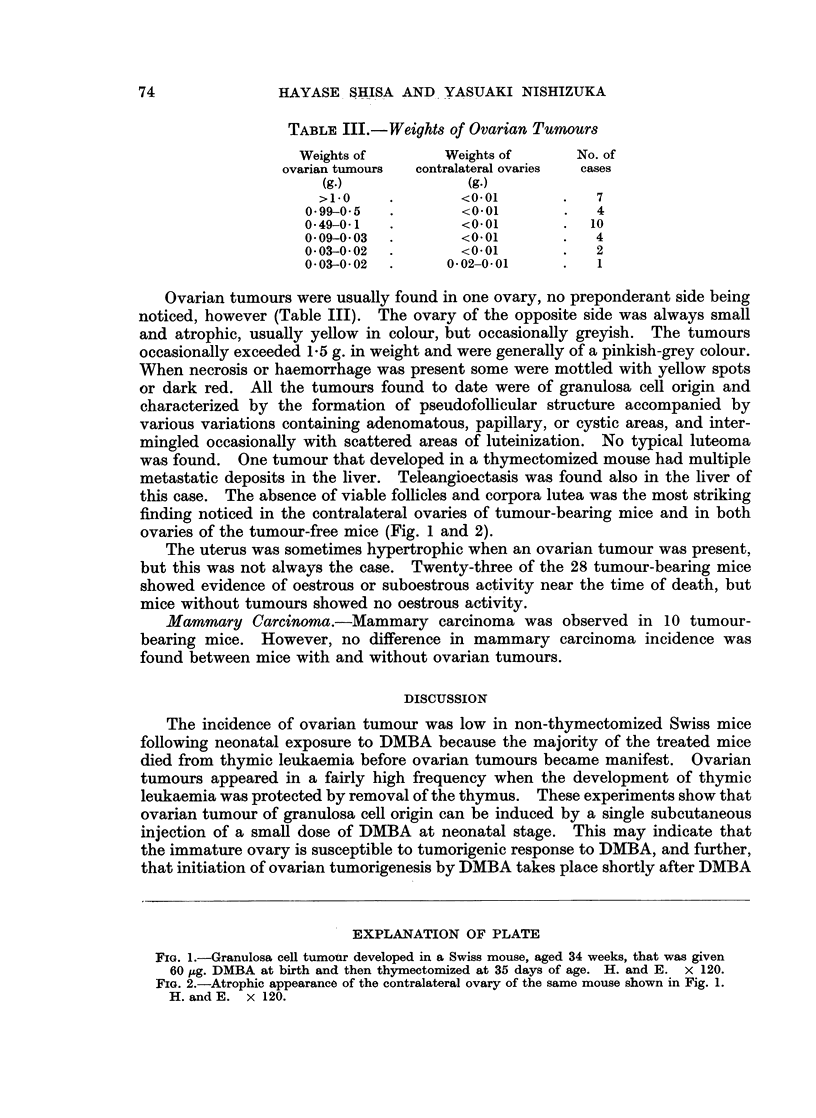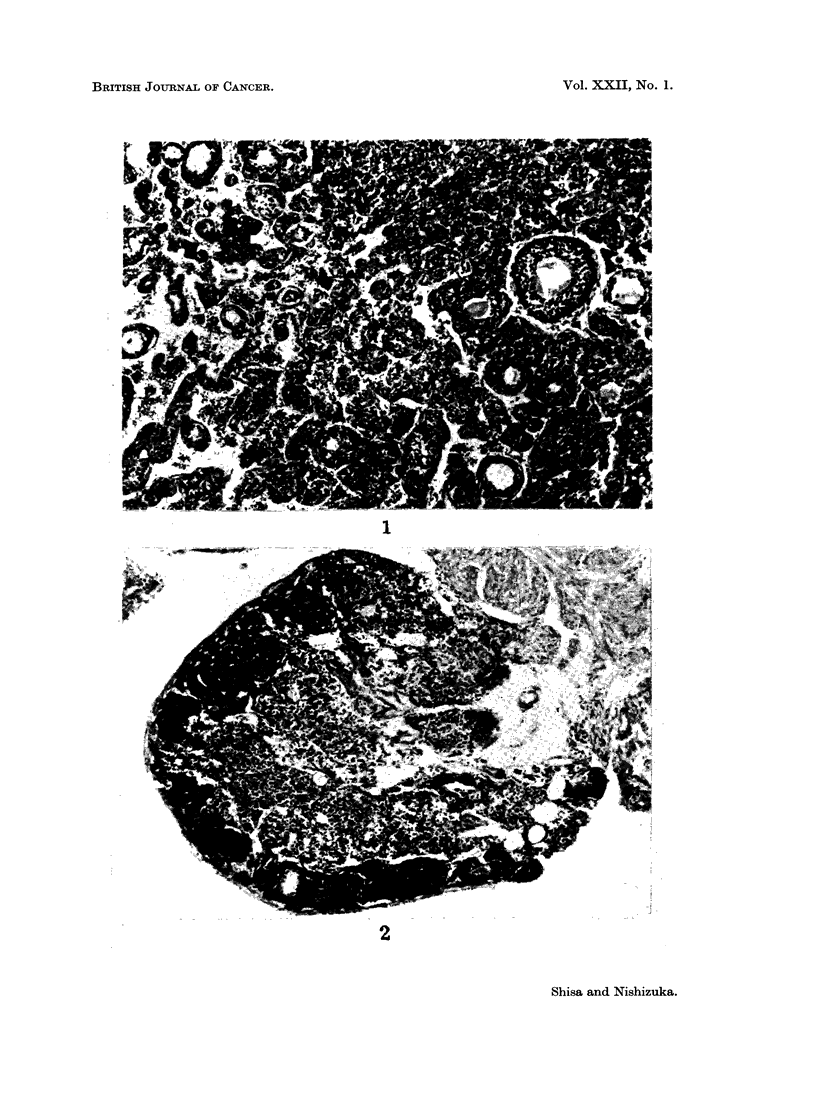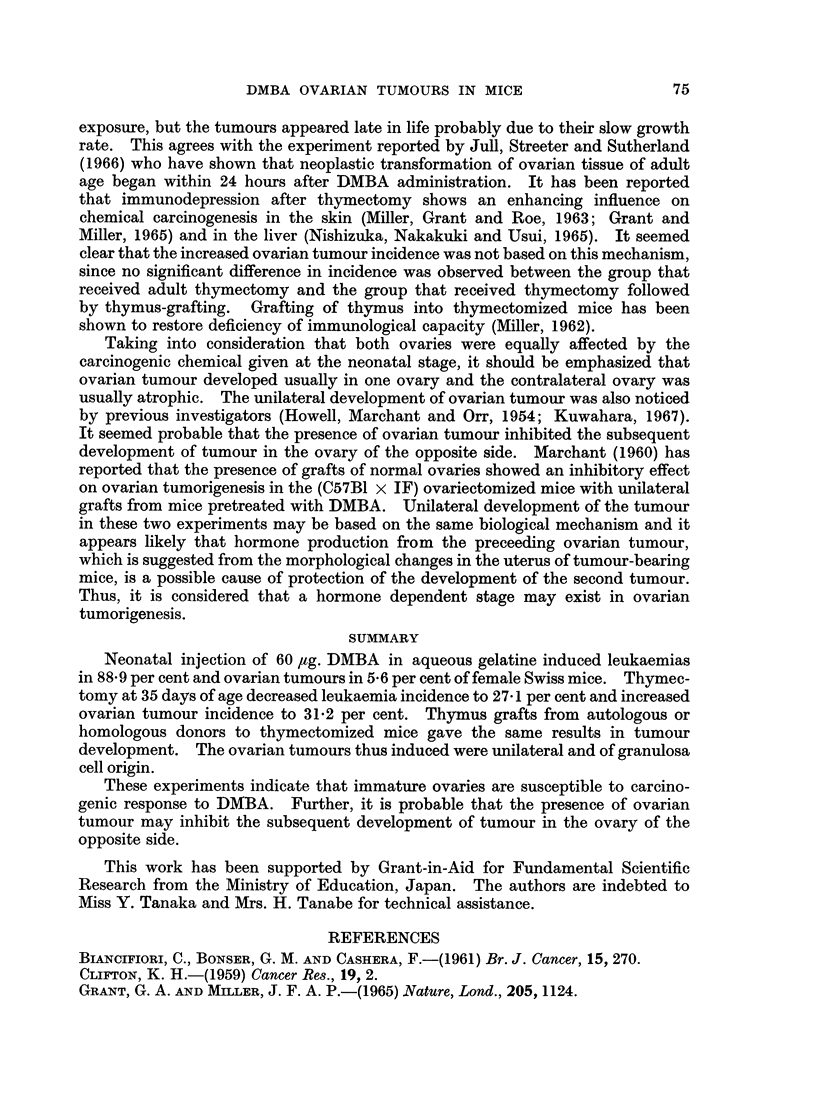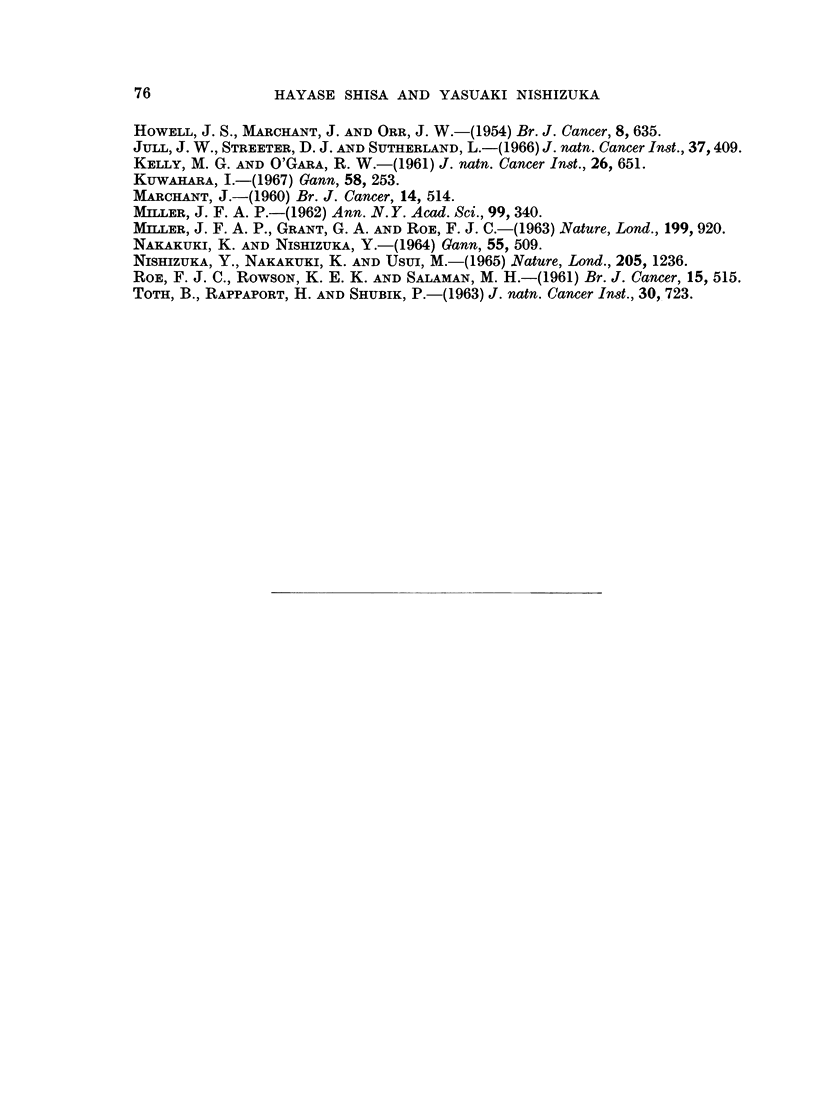# Unilateral development of ovarian tumour in thymectomized Swiss mice following a single injection of 7,12-dimethylbenz(a)anthracene at neonatal stage.

**DOI:** 10.1038/bjc.1968.10

**Published:** 1968-03

**Authors:** H. Shisa, Y. Nishizuka

## Abstract

**Images:**


### UNILATERAL DEVELOPMENT OF OVARIAN TUMOUR IN THYMECTOMIZED SWISS MICE FOLLOWING A SINGLE INJECTION OF 7,12-DIMETHYLBENZ(*a*) ANTHRACENE AT NEONATAL STAGE

HAYASE SHISA AND YASUAKI NISHIZUKA

*From the Laboratory of Experimental Pathology, Aichi Cancer Center Research Institute, Chikusa-ku, Nagoya, Japan*

Received for publication September 18, 1967

OVARIAN tumours in the mouse can be induced by different experimental procedures (reviewed by Clifton, 1959). Howell, Marchant and Orr (1954) and Biancifiori, Bonser and Caschera (1961) reported that a high incidence of ovarian granulosa cell tumours appeared in mice following repeated cutaneous application or oral administration of 7,12-dimethylbenz(*a*) anthracene (DMBA). Recently, Jull, Streeter and Sutherland (1966) and Kuwahara (1967) noted that a single application of DMBA by oral and intraveneous routes is effective in inducing ovarian tumours in young adult mice. It is now well known that DMBA is a very potent carcinogen capable of eliciting various types of neoplasms in mice, given as newborns (Kelly and O'Gara, 1961; Roe, Rowson and Salaman, 1961; Toth, Rappaport and Shubik, 1963). Thymic leukaemia is the tumour that appears most frequently in younger ages in Swiss mice neonatally treated with DMBA. Thus, it is possible to postulate that if the appearance of leukaemia could be prevented, other types of tumours might appear in fairly high frequency in the DMBA-exposed Swiss mice. Thymectomy is shown to be effective in preventing the development of DMBA-induced leukaemia in Swiss mice. The present report concerns unilateral development of ovarian tumours in the thymectomized Swiss mice that received a single injection of DMBA, in a small dose, at neonatal age.

### EXPERIMENTAL PROCEDURES

Female Swiss mice, raised in our laboratory, were used in the present experiment. They were given one subcutaneous injection, at the interscapular region, of 60 $\mu$g. of DMBA, suspended in 1·0 per cent aqueous gelatine solution, at birth (within 24 hours after birth). Mice of Group I were kept without any further treatment. Neonatally DMBA-treated mice of Group II received thymectomy at 35 $\pm$ 5 days of age under the technique previously described (Nakakuki and Nishizuka, 1964) to prevent leukaemia development. Mice of Group III were given DMBA injection at birth, then thymectomized at 35 $\pm$ 5 days of age and grafted subcutaneously either with 1-day-old homologous (Swiss) thymuses or with autologous thymuses; each animal received one whole thymus. The thymus grafts were placed into the inguinal fat pads of the right side immediately after thymectomy. Many grafts were recovered when the mice were killed.

Female Swiss mice without any treatment and mice thymectomized at 35 $\pm$ 5 days with or without grafting of 1-day-old whole thymus of Swiss mice served as controls.

The mice were kept in metal boxes, 5–6 per box, and were fed on a commercial diet in pellet form. They were inspected twice a week for leukaemia and other tumours and were killed when leukaemia became evident or palpable tumours became large or they were otherwise in poor condition. At autopsy, solid ovarian tumours or cysts, and the uterus as well, were removed, weighed on a torsion balance and examined histologically. Leukaemic tissues, lung, kidney, liver, uteri, and ovaries with normal appearance were also removed for histological study. Most of the animals that reached 34 weeks of age were killed since they were usually in poor conditions due to the development of multiple lung adenomatosis, and only a few animals were allowed to live until they reached 52 weeks of age.

### EXPERIMENTAL FINDINGS

As shown in Tables I and II, control Swiss mice without DMBA treatment had low incidence of leukaemia and ovarian tumour. DMBA treatment at neonatal stage yielded a high incidence of thymic leukaemia. Thymectomy performed at 35 days of age resulted in a marked reduction in leukaemic incidence of the Swiss mice neonatally injected with DMBA. The grafting of thymic tissue, homologous and autologous, did not restore leukaemia development in the present experimental system. The survival of mice of the three experimental groups, non-thymectomized, thymectomized, and thymectomized followed by thymus grafting, was in general determined by the rate of appearance of leukaemia. Animals killed or that died after 15 weeks of age always had multiple lung adenomas of varying numbers and sizes. Table I gives incidence of leukaemias and other tumours in all the groups, and Table II, the incidence of ovarian tumours.

*Leukaemia.*—Thymic leukaemia developed in 46 out of the 54 mice (88·9 per cent) in Group I. Deaths from leukaemia commenced at 8 weeks of age and the majority of leukaemic mice died at less than 26 weeks of age. In contrast, the incidence of leukaemia in both Groups II and III fell into a range of 27–29 per cent, and about half of leukaemic cases died at the ages over 26 weeks after DMBA injection. The details of leukaemogenesis in thymectomized mice will be described elsewhere.

*Ovarian Tumour.*—Only 3 of the total of 54 females of Group I developed ovarian tumours, the incidence being only 5·6 per cent with a mean latency of 29 weeks. In Groups II and III, where leukaemia development was markedly inhibited by thymectomy, the incidence of ovarian tumours increased and was 31·2 per cent and 28·6 per cent, respectively. Of the total of 83 thymectomized mice, macroscopic tumours were present in 23 animals. In 2 mice, tumours were suspected macroscopically because of inequality in size of the ovaries, though the larger ovary did not exceed 30 mg. in weight. Inequality in size of the ovaries was occasionally observed and some of the larger ovaries contained proliferative foci composed of darker cells probably showing pseudofollicular differentiation. However, because accurate recognition of an early tumour offered difficulties, diagnosis of tumour was mostly based upon the size of enlarged ovary. Ovaries less than 20 mg. irrespective of the presence of histologic patterns suggestive of neoplastic growth has been excluded from the data in Table II. The earliest ovarian tumour was found in a thymectomized mouse of 17 weeks of age. The mean latent period of the tumour development was almost the same in the two thymectomized groups.

TABLE I.—*Incidence of Leukaemia and Other Tumours in Non-thymectomized and Thymectomized Female Swiss Mice Given* 60 *μg. DMBA at Birth*

| Group | DMBA treatment | Thymectomy and thymus grafting | No. of mice | Leukaemia <26 wks | Leukaemia >26 wks | Leukaemia Total | Mammary tumour | Lung adenoma | Other tumours |
|---|---|---|---|---|---|---|---|---|---|
| Control . | No | no | 59 | 1 | 1 | 2 | 2 | 18 (1·2)* | Papilloma of skin 1 |
| „ . | No | Thymectomy at 35 d. of age | 18 | 0 | 1 | 1 | 1 | 8 (1·7) | Liver tumour 1 |
| „ . | No | Thymectomy and 1 day thymus graft at 35 d. of age | 27 | 0 | 2 | 2 | 1 | 11 (1·7) | Liver tumour 1. Fibroma of parotid 1 |
| Group I . | Yes | no | 54 | 46 | 2 | 48 (88·9%) | 0 | 52 (19·9) | — |
| Group II . | Yes | Thymectomy at 35 d. of age | 48 | 8 | 5 | 13 (27·1%) | 2 | 47 (18·8) | Fibroma of uterus 1. Papilloma of ear duct 1. Subcutaneous sarcoma 2† |
| Group III | Yes | Thymectomy and 1 day thymus graft at 35 d. of age | 21 | 3 | 3 | 6 | 1 | 21 | — |
| Group III | Yes | Thymectomy and autologous thymus graft at 35 d. of age | 14 | 2 | 2 | 4 | 1 | 14 | — |
| Group III | | Total | 35 | 5 | 5 | 10 (28·6%) | 2 | 35 (22·1) | |

* Mean number of nodules of lung adenomatosis per mouse

† Sarcoma developed at the site of DMBA injection

TABLE II.—*Incidence of Ovarian Tumour in Non-thymectomized and Thymectomized Swiss Mice Given* 60 $\mu g$. *DMBA at Birth*

| Group | DMBA treatment | Thymectomy and thymus grafting | No. of mice | Ovarian tumours <26 wks | <34 wks | <42 wks | >43 wks | Total | Average latency |
|---|---|---|---|---|---|---|---|---|---|
| Control . | No | no | 59 | 0 | 0 | 1 | 0 | 1 | 42 wks |
| ,, . | No | Thymectomy at 35 d. of age | 18 | 0 | 0 | 0 | 0 | 0 | — |
| ,, . | No | Thymectomy and 1 day thymus graft at 35 d. of age | 27 | 0 | 0 | 0 | 0 | 0 | — |
| Group I . | Yes | no | 54 | 1 | 2 | 0 | 0 | 3 (5·6%) | 29 wks |
| Group II . | Yes | Thymectomy at 35 d. of age | 48 | 2 | 5 | 5 | 3 | 15 (31·2%) | 35·8 wks |
| Group III | Yes | Thymectomy and 1 day thymus graft at 35 d. of age | 21 | 1 | 1 | 1 | 3 | 6 | — |
| Group III | Yes | Thymectomy and autologous thymus graft at 35 d. of age | 14 | 2 | 2 | 0 | 0 | 4 | — |
| Group III | | Total | 35 | 3 | 3 | 1 | 3 | 10 (28·6%) | 35·8 wks |

TABLE III.—*Weights of Ovarian Tumours*

| Weights of ovarian tumours (g.) | Weights of contralateral ovaries (g.) | No. of cases |
|---|---|---|
| >1·0 | <0·01 | 7 |
| 0·99–0·5 | <0·01 | 4 |
| 0·49–0·1 | <0·01 | 10 |
| 0·09–0·03 | <0·01 | 4 |
| 0·03–0·02 | <0·01 | 2 |
| 0·03–0·02 | 0·02–0·01 | 1 |

Ovarian tumours were usually found in one ovary, no preponderant side being noticed, however (Table III). The ovary of the opposite side was always small and atrophic, usually yellow in colour, but occasionally greyish. The tumours occasionally exceeded 1·5 g. in weight and were generally of a pinkish-grey colour. When necrosis or haemorrhage was present some were mottled with yellow spots or dark red. All the tumours found to date were of granulosa cell origin and characterized by the formation of pseudofollicular structure accompanied by various variations containing adenomatous, papillary, or cystic areas, and intermingled occasionally with scattered areas of luteinization. No typical luteoma was found. One tumour that developed in a thymectomized mouse had multiple metastatic deposits in the liver. Teleangioectasis was found also in the liver of this case. The absence of viable follicles and corpora lutea was the most striking finding noticed in the contralateral ovaries of tumour-bearing mice and in both ovaries of the tumour-free mice (Fig. 1 and 2).

The uterus was sometimes hypertrophic when an ovarian tumour was present, but this was not always the case. Twenty-three of the 28 tumour-bearing mice showed evidence of oestrous or suboestrous activity near the time of death, but mice without tumours showed no oestrous activity.

*Mammary Carcinoma.*—Mammary carcinoma was observed in 10 tumour-bearing mice. However, no difference in mammary carcinoma incidence was found between mice with and without ovarian tumours.

### DISCUSSION

The incidence of ovarian tumour was low in non-thymectomized Swiss mice following neonatal exposure to DMBA because the majority of the treated mice died from thymic leukaemia before ovarian tumours became manifest. Ovarian tumours appeared in a fairly high frequency when the development of thymic leukaemia was protected by removal of the thymus. These experiments show that ovarian tumour of granulosa cell origin can be induced by a single subcutaneous injection of a small dose of DMBA at neonatal stage. This may indicate that the immature ovary is susceptible to tumorigenic response to DMBA, and further, that initiation of ovarian tumorigenesis by DMBA takes place shortly after DMBA

---

### EXPLANATION OF PLATE

FIG. 1.—Granulosa cell tumour developed in a Swiss mouse, aged 34 weeks, that was given 60 μg. DMBA at birth and then thymectomized at 35 days of age. H. and E. × 120.

FIG. 2.—Atrophic appearance of the contralateral ovary of the same mouse shown in Fig. 1. H. and E. × 120.

1

2

exposure, but the tumours appeared late in life probably due to their slow growth rate. This agrees with the experiment reported by Jull, Streeter and Sutherland (1966) who have shown that neoplastic transformation of ovarian tissue of adult age began within 24 hours after DMBA administration. It has been reported that immunodepression after thymectomy shows an enhancing influence on chemical carcinogenesis in the skin (Miller, Grant and Roe, 1963; Grant and Miller, 1965) and in the liver (Nishizuka, Nakakuki and Usui, 1965). It seemed clear that the increased ovarian tumour incidence was not based on this mechanism, since no significant difference in incidence was observed between the group that received adult thymectomy and the group that received thymectomy followed by thymus-grafting. Grafting of thymus into thymectomized mice has been shown to restore deficiency of immunological capacity (Miller, 1962).

Taking into consideration that both ovaries were equally affected by the carcinogenic chemical given at the neonatal stage, it should be emphasized that ovarian tumour developed usually in one ovary and the contralateral ovary was usually atrophic. The unilateral development of ovarian tumour was also noticed by previous investigators (Howell, Marchant and Orr, 1954; Kuwahara, 1967). It seemed probable that the presence of ovarian tumour inhibited the subsequent development of tumour in the ovary of the opposite side. Marchant (1960) has reported that the presence of grafts of normal ovaries showed an inhibitory effect on ovarian tumorigenesis in the (C57Bl $\times$ IF) ovariectomized mice with unilateral grafts from mice pretreated with DMBA. Unilateral development of the tumour in these two experiments may be based on the same biological mechanism and it appears likely that hormone production from the preceeding ovarian tumour, which is suggested from the morphological changes in the uterus of tumour-bearing mice, is a possible cause of protection of the development of the second tumour. Thus, it is considered that a hormone dependent stage may exist in ovarian tumorigenesis.

### SUMMARY

Neonatal injection of 60 $\mu$g. DMBA in aqueous gelatine induced leukaemias in 88·9 per cent and ovarian tumours in 5·6 per cent of female Swiss mice. Thymectomy at 35 days of age decreased leukaemia incidence to 27·1 per cent and increased ovarian tumour incidence to 31·2 per cent. Thymus grafts from autologous or homologous donors to thymectomized mice gave the same results in tumour development. The ovarian tumours thus induced were unilateral and of granulosa cell origin.

These experiments indicate that immature ovaries are susceptible to carcinogenic response to DMBA. Further, it is probable that the presence of ovarian tumour may inhibit the subsequent development of tumour in the ovary of the opposite side.

This work has been supported by Grant-in-Aid for Fundamental Scientific Research from the Ministry of Education, Japan. The authors are indebted to Miss Y. Tanaka and Mrs. H. Tanabe for technical assistance.

### REFERENCES

Biancifiori, C., Bonser, G. M. and Cashera, F.—(1961) *Br. J. Cancer*, **15**, 270.
Clifton, K. H.—(1959) *Cancer Res.*, **19**, 2.
Grant, G. A. and Miller, J. F. A. P.—(1965) *Nature, Lond.*, **205**, 1124.

Howell, J. S., Marchant, J. and Orr, J. W.—(1954) *Br. J. Cancer*, **8,** 635.
Jull, J. W., Streeter, D. J. and Sutherland, L.—(1966) *J. natn. Cancer Inst.*, **37,** 409.
Kelly, M. G. and O'Gara, R. W.—(1961) *J. natn. Cancer Inst.*, **26,** 651.
Kuwahara, I.—(1967) *Gann*, **58,** 253.
Marchant, J.—(1960) *Br. J. Cancer*, **14,** 514.
Miller, J. F. A. P.—(1962) *Ann. N.Y. Acad. Sci.*, **99,** 340.
Miller, J. F. A. P., Grant, G. A. and Roe, F. J. C.—(1963) *Nature, Lond.*, **199,** 920.
Nakakuki, K. and Nishizuka, Y.—(1964) *Gann*, **55,** 509.
Nishizuka, Y., Nakakuki, K. and Usui, M.—(1965) *Nature, Lond.*, **205,** 1236.
Roe, F. J. C., Rowson, K. E. K. and Salaman, M. H.—(1961) *Br. J. Cancer*, **15,** 515.
Toth, B., Rappaport, H. and Shubik, P.—(1963) *J. natn. Cancer Inst.*, **30,** 723.